# Specialisation among Hospitals

**Published:** 1909-12-04

**Authors:** 


### Specialisation Among Hospitals.

A circular has lately been printed by the management of the National Hospital for Diseases of the Heart, Soho Square, which is signed by all the honorary physicians of the staff. The pamphlet, which is very ably drawn up, sets out to prove the need for this prticular institution, and to explain why it is that cases of heart disease are unsuitable for treatment in a general hospital. Since this is quite frankly the object of the circular, the arguments adduced merit close consideration.

Before, however, turning to an analysis of the thesis thus advanced it is not irrelevant to dwell for a moment on the present treatment of heart cases and to point out one or two obvious facts with which the pamphlet does not deal. First, this hospital contains but 26 beds, and it is the only special hospital for diseases of the heart in London. Either, therefore, the whole of the London hospital world is doing that which the memorandum teaches is woefully wrong, and the needs of Londoners are disgracefully neglected; or else the case as therein developed contains a serious flaw. The former alternative condemns by implication the wisdom and the humanity of every single one of the leaders of the profession, and for reasons to be presently shown it is probable that the latter is the more nearly correct. Again, the fact that in general hospitals out-patients are usually treated by members of the staff who have few, if any, in-patient beds, cannot really militate against that careful study of cases necessary for the making of a clinician; for Sibson, Broadbent, Wilks, and scores of others, who have contributed largely to the establishment of modern views on heart disease, have been physicians to general hospitals. Finally, in Edinburgh, where special hospitals flourish even more abundantly (in proportion) than in the metropolis, it has not yet been thought necessary to establish a separate hospital for diseases of the heart; and we are not able to recall any other town in Britain of which the same cannot be said.

As for the pamphlet itself, at first all is sweet reasonableness. It is pointed out that in connection with certain regions—the eye for instance—the degree of proficiency required in the use of special instruments practically compels the abandonment of general medicine and surgery by those who devote themselves to its acquisition. This is indisputable, though it by no means follows that because we must have specialist ophthalmologists we must also have special hospitals for them to practise in. The other class of special hospital, proceed the authors, is required from the special circumstances of the diseases treated rather than from special qualities demanded of the medical staff. Infectious fevers furnish an apt case in point. Agreed; and since tuberculosis is a specific infectious fever, the argument can rightly be extended to hospitals for consumption. The next development is the exclusion of cardiac patients from consumption hospitals, which follows quite logically; but the fallacy arises when it is deduced from this that special heart hospitals are therefore necessary. We cannot admit this conclusion, because we dissent entirely from the premiss that general hospitals are unsuitable for the treatment of diseases of the heart.

If heart cases are to be excluded from general hospitals, and this is the necessary result of adopting the principles advocated in the circular, it is difficult to see what there will be left for general hospitals to do; for every other disease is equally entitled to special consideration. To state this is to show that specialism is here running riot indeed, and if the argument is pushed right home it must be extended to finer sub-division still, and one hospital must treat aortic, and another mitral diseases, and a third the two combined. Perhaps the most convincing answer to the demand of Drs. Chapman, Gibbes, Moon, and Wells is to point out that when the heart is diseased there is not a single organ in the body which does not suffer ill-effects, and that, therefore, a general hospital is the proper place for a patient with heart disease and a general physician the proper person to treat him.

